# Incidence Rates of Breast Cancer by Age and Tumor Characteristics Among Saudi Women: Recent Trends

**DOI:** 10.7759/cureus.6664

**Published:** 2020-01-15

**Authors:** Sulaiman Asiri, Amira Asiri, Sibi Ulahannan, Mokhlef Alanazi, Abdullah Humran, Abdulelah Hummadi

**Affiliations:** 1 Surgery, Najran University, Najran, SAU; 2 Surgery, King Khalid University, Khamis Mushait, SAU; 3 Biostatistics, Continuous Quality Improvement & Patient Safety, Armed Forces Hospital Southern Region, Khamis Mushait, SAU; 4 General Surgery, Armed Forces Hospital Southern Region, Khamis Mushait, SAU

**Keywords:** breast cancer, saudi women, incident rate, saudi arabia

## Abstract

Introduction and objectives

With such a huge country as Saudi Arabia, it would be expected to have variations in the pattern and incidence of breast diseases. This study aims to determine the recent trends in breast cancer incidence rates by age and tumor characteristics among female patients treated in the Armed Forces Hospital Southern Region (AFHSR) from the period of January 2010 to December 2017.

Methods

This study is a retrospective chart review where all breast biopsy reports of female patients were seen between January 2010 and December 2017 at the AFHSR, Saudi Arabia, to observe the pattern of breast cancer as well as to calculate the incidence rates by age and tumor characteristics among the study subjects.

Results

Overall, the incidence rates of breast cancer among female patients ranged between three to eight confirmed cases of breast cancer for every 1000 patients for the period of 2010 to 2017, where the highest incidence rate was reported in the year of 2017. Additionally, two distinct patterns are observed in breast cancer trends, i.e., the most common type of cancer was ductal carcinoma with an incidence percentage of 81.80%, followed by lobular carcinoma (3.40%). There was no statistical evidence that the associated population means of age are significantly different from the type of tumor characteristics.

Conclusions

In this study, the average age for diagnosed women with invasive breast cancer is about 56 years of age while in situ is 51 years. Among women of all age groups, ductal carcinoma is the most common. There is also an increase in the incidence of breast cancer between 2016 to 2017, where the highest incidence rate was reported in the year 2017. Continued vigilance, mammographic screening, and patient education are needed to establish an early diagnosis and perform the optimal treatment.

## Introduction

It is important to emphasize that breast cancer remains the leading cause of death among Saudi women [1]. Breast cancer is not only a significant problem in Saudi Arabia but also considered to be one of the most common causes of cancer-related mortality worldwide [2]. In order to most appropriately allocate healthcare and research funding for cancer, it is crucial to have accurate population-based incidence data [3]. Breast cancer (BC) has a significant impact on the health of women worldwide, and the Kingdom of Saudi Arabia (KSA) is no exception [4].

According to the Saudi Cancer Registry (SCR) statistics, the total number of cases of cancer identified by the SCR in 2012 was 14,336, with 6,791 (47.5%) among males and 7,545 (52.6%) among females [3]. In 2014, breast cancer ranked first among females, where there were 1,826 female breast cancer cases between January and December 2014. Breast cancer accounted for 15.9% of all cancers reported among Saudi nationals and for 28.7% of all cancers reported among females of all ages. The age-standardized rate (ASR) was 22.7/100,000 for the female population while, at diagnosis, the median age was 50 years [5].

Additionally, it is essential to highlight the fact that breast cancer remains the leading cause of death among Saudi women [1]. Breast disease outlines are still not well-reported in Saudi Arabia [6]. The current situation of this frequent and severe health problem is somehow less than ideal, which indicates that further efforts need to be made to detect the disease at an earlier stage [6]. This can be achieved by encouraging more extensive and confident use of fine-needle aspiration (FNA) in the routine practice of palpable breast lesions [6]. In this study, 296 surgical breast biopsies and mastectomies were collected from the department of surgical pathology, Armed Force Hospital Southern Region (AFHSR), Saudi Arabia. This study aims to determine the recent trends in breast cancer incidence rates by age and tumor characteristics among female patients treated in the AFHSR from the period of January 2010 to December 2017.

## Materials and methods

This study is a retrospective chart review that took place in the Armed Force Hospital Southern Region. It covered Saudi female patients only; we didn’t exclude any age group but excluded benign cases. Data were collected regarding the age of diagnosis and the histopathological report. The age was divided into eight groups with a five-year interval (<40, 40-45, 46-50, 51-55, 56-60, 61-65, 66-70, >70) where we got the trend of the incidence of breast cancer by age at the time of diagnosis. The tumor characteristics were divided into ductal carcinoma in situ, lobular carcinoma in situ, invasive ductal carcinoma, invasive lobular carcinoma, mucinous (colloid) carcinoma, invasive mammary carcinoma, and other, i.e., confirmed breast cancer but histopathological non-specified. After that, we got the trend of incidence of breast cancer by tumor characteristics through the histopathological report at the time of diagnosis. The study has some limitations as a result of its retrospective nature. First is the fact that it was conducted at a single center with relatively small sample size; future research should cover a larger sample size. In addition, it used the cohort methodology in order to develop country-specific clinical practice guidelines, which are necessarily required and will address the issues associated with screening programs.

## Results

**Figure 1:**
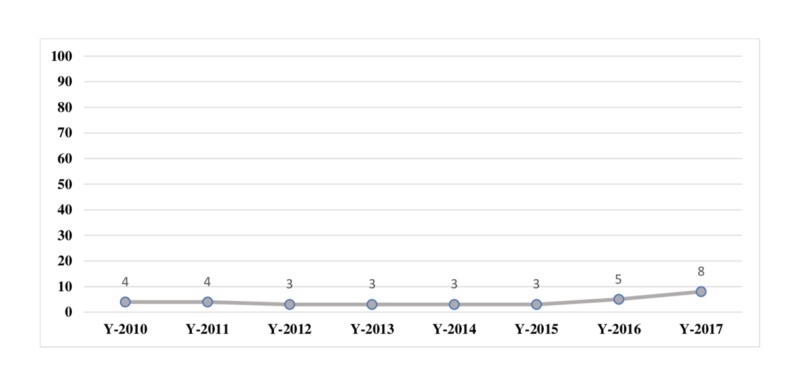
Incidence rate of female breast cancer per 1000 patients from 2010 to 2017

Overall, the incidence rates of breast cancer among female patients ranged between three to eight confirmed cases of breast cancer for every 1,000 patients for the period of 2010 to 2017, where the highest incidence rate was reported in the year 2017 (Table 1, Figure 1).

**Table 1:** Type of tumor with average age at diagnosis

Statistics-Age at Diagnosis	Overall Sample	Invasive	In Situ
Number of samples (frequency)	296	233	19
Mean	55.93	55.68	50.78
Median	54.00	54.00	43.00
Std. deviation	15.96	15.63	15.50
Minimum	28.00	28.00	33.00
Maximum	92.00	92.00	91.00
Percentage (%)		92.46%	7.54%

Furthermore, two distinct patterns are observed in breast cancer trends: the most common type of cancer was ductal carcinoma, with a percentage of 81.80%, followed by lobular carcinoma (3.40%). There was no statistical evidence that the associated population means of age are significantly different from the types of tumor characteristics.

Moreover, when inspecting all the breast lesions for the time frame of seven years, we came across a total of 296 cases, which were filtered and categorized into seven main groups: first, ductal carcinoma, 242 cases (81.80%); second, lobular carcinoma 10 cases (3.40%); third, invasive mucinous carcinoma, eight cases (2.70%); fourth, invasive mammary carcinoma, three cases (1.00%); fifth, basal cell carcinoma, three cases (1.00%); sixth, adenocarcinoma, one case (0.30%); seventh, the other 22 cases (9.80%). The mean age for these groups was 55.49, 51.20, 60.50, 69.33, 83.00, 82.0, and 54.90 years, respectively (Table 2).

**Table 2:** Incidence rate of female breast cancer per 1000 patients from 2010 to 2017 AFHSR: Armed Forces Hospital Southern Region

Year	Y-2010	Y-2011	Y-2012	Y-2013	Y-2014	Y-2015	Y-2016	Y-2017
Breast Cancer Confirmed Cases	31	31	29	32	29	28	47	69
All Visits to AFHSR General Surgery Clinic	8197	8232	9251	9304	9402	9570	9817	8947
Incidence Rate Per 1000 Patients	4	4	3	3	3	3	5	8

**Figure 2:**
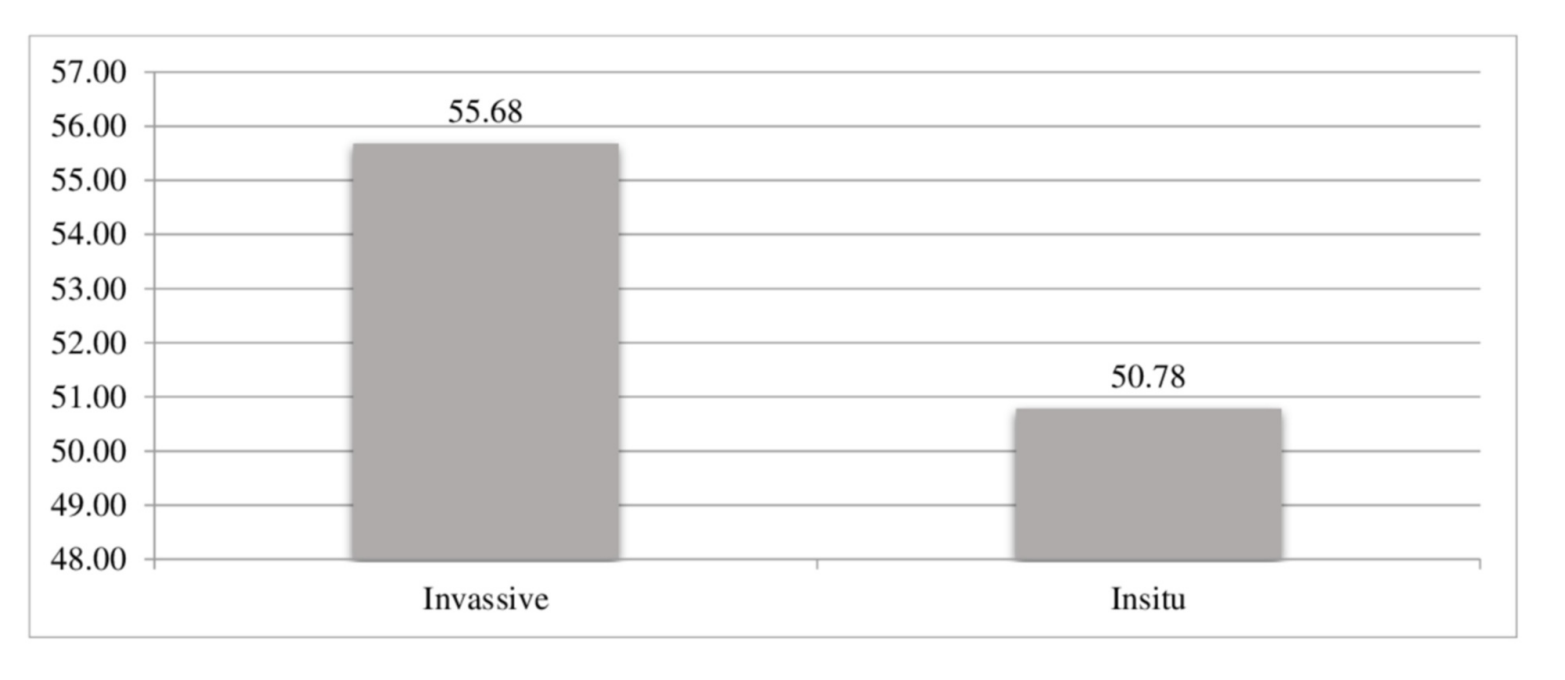
Type of tumor with average age at diagnosis

In addition, the average age at diagnosis for the invasive group was high (55.68) as compared to the in situ group (50.78) ) as shown in Figure 2, but there was no statistical evidence that the associated population means of age are significantly different with the types of tumor characteristics.

**Figure 3:**
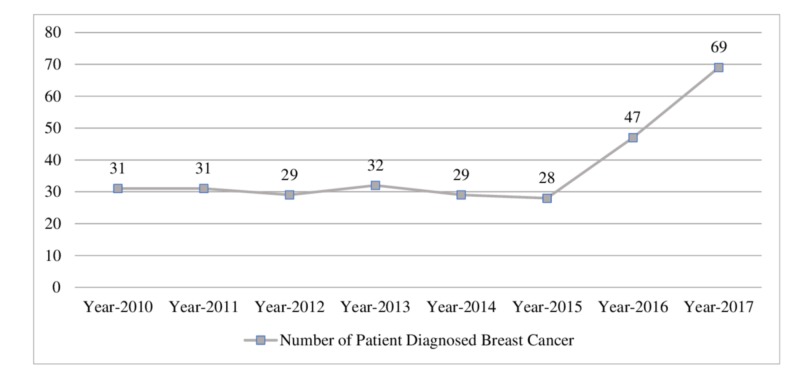
Total number of patients diagnosed with breast cancer per year

The number of women with breast cancer sharply increased from 2016 to 2017. This increase may be explained by the growth of the use of mammography and the effectiveness of the breast cancer awareness program that has been initiated by the government. An additional factor is a campaign organized by numerous health authorities in order to educate the public on the early detection of breast cancer through a self-breast examination (Table 3, Figure 3).

**Table 3:** Total number of patients diagnosed with breast cancer per year

Statistics-Age at Diagnosis	Ductal Carcinoma	Lobular Carcinoma	Invasive Mucinous Carcinoma	Invasive Mammary Carcinoma	Adenocarcinoma	Basal Cell Carcinoma	Others
Number of sample	242	10	8	3	1	3	29
Mean	55.49	51.20	60.50	69.33	82.00	83.00	54.90
Median	53.00	53.00	67.00	73.00	82.00	83.00	51.00
Std. deviation	15.99	5.83	18.09	21.73		0.00	14.84
Minimum	28.00	41.00	34.00	46.00	82.00	83.00	34.00
Maximum	92.00	60.00	83.00	89.00	82.00	83.00	88.00

According to the findings, the age groups that were least affected by breast cancer were women aged from 66 to 70 years, followed by those aged 56-60 years. Moreover, the group of older Saudi women, those aged above 70 years, recorded the highest overall number. Overall, the most commonly reported malignant lesion was ductal carcinoma: 242 cases (81.80%), followed by lobular carcinoma: 10 cases (3.40%), and invasive mucinous carcinoma: eight cases (2.70%) (Table 4).

**Table 4:** Average age of patients at diagnosis

Year	Year-2010	Year-2011	Year-2012	Year-2013	Year-2014	Year-2015	Year-2016	Year-2017
Number of Patient Diagnosed Breast Cancer	31	31	29	32	29	28	47	69
Average Age at Diagnosis	54.74	62.23	54.48	56.38	57.41	50.79	57.47	54.45

Significant fractions of our patient collection (mean age 55.49 years) with malignant lesions had ductal carcinoma (81.80%) as the most commonly reported tumor (242 cases). The second-ranking lesion was lobular carcinoma comprising 10 (3.40%) cases (mean age 51.20 years) and the third was invasive mucinous carcinoma, three (60.50) cases (Table 2).

Out of these 233 cases, there were invasive cancers and 19 were intraductal carcinomas. The mean age of invasive cancers and in situ was 55.68 and 50.78 years, respectively (Table 4).

## Discussion

The present study highlighted the recent trends in breast cancer incidence rates by age and tumor characteristics among Saudi women. As a retrospective hospital laboratory-based study, it has the limitation of dependence on the data-collecting efficacy and performance of other personnel of the hospital. Moreover, a critical statistical analysis could not be performed due to the non-availability of appropriate comparative figures. However, it has served the purpose of providing primary data, which is comparable with similar recent and historical studies. The patterns of incidence and mortality rates for breast cancer vary across countries and are attributable to a combination of demographic, heredity, environmental, and lifestyle factors [7]. In developing countries, the incidence of breast cancer is increasing and is becoming more similar to developed countries [7]. Although, at present, the incidence of breast cancer in developed countries remains higher, mortality is lower [7]. Several factors, including late presentation, may explain this, as the disease stage at diagnosis is strongly associated with patient outcomes [7].

Current guidelines from the Saudi Center of Evidence-Based Healthcare recommend starting screening with mammography at the age of 40, five years younger than the American Cancer Society guidelines [7]. This recommended age was chosen by the Saudi expert panel based on the increased incidence of breast cancer in patients aged 40-49 years as compared with other countries [8-9]. Although the average age of diagnosis falls within this age group (40-49 years), 20% of our study population was younger and would not have been diagnosed with screening. Furthermore, the application of these guidelines has been limited, as indicated by another study that found that almost 92% of women over 50 have never undergone screening [7]. According to current guidelines, patients aged >40 years should undergo mammography every one or two years. However, cancers are more likely to be diagnosed by the detection of a palpable mass than by screening [7]. One reason for the increasing incidence of breast cancer in developed countries could be the aging population [7]. For example, the average patient age in Sweden is 60 years and in Mexico, it is 50 years, representing a 10-year difference [7]. Our study population had a predominance of young patients (61.7% of patients were younger than 50 years), which is expected for this region; the average age in previous studies ranged between 40 and 45 years [7]. It is additionally vital to highlight the fact that breast cancer remains the leading cause of death among Saudi women [1]. In this study, the average age at presentation of breast cancer is shown to be six years older than the national average and nine years older than the Arabian average [10]. This data suggests that there may be clear population differences in the age of presentation of breast cancer between Saudi and Arab populations. The effect of such differences could result in significant changes in disease presentation and behavior. Screening would not benefit the patient if not followed by treatment (including surgery), and treatment is expected to be more effective if cancer is detected at an earlier stage by screening. As per the study of Berry and colleagues, the combination of treatment following screening has an overall estimated reduction in breast cancer death from 24.9%-38.3%, where breast screening contributes to this reduction with 28% to 65% (median, 46%) [11]. The importance of mammographic breast screening is clear, as it currently is probably the most critical approach to decrease mortality from breast cancer [10].

## Conclusions

In this study, the average age for diagnosed women with invasive breast cancer is about 56 years of age while in situ is 51 years. This age is six years older than the national average and nine years older than the Arabian average. Among all age group women, ductal carcinoma is the most common. Among the study population, there is an increase in the incidence of breast cancer between 2016 and 2017. The highest incidence rate reported was in the year 2017, with eight confirmed cases of breast cancer per every 1000 patients. Continued vigilance, mammographic screening, and patient education are needed to establish an early diagnosis and perform the optimal treatment.
